# Oral health-related quality of life in patients with early rheumatoid arthritis is associated with periodontal inflammation and painful temporomandibular disorders: a cross-sectional study

**DOI:** 10.1007/s00784-021-04034-z

**Published:** 2021-07-19

**Authors:** Johanna M. Kroese, Catherine M. C. Volgenant, Dirkjan van Schaardenburg, Laurette van Boheemen, Maurits K. A. van Selms, Corine M. Visscher, Wim Crielaard, Bruno G. Loos, Frank Lobbezoo

**Affiliations:** 1 Department of Orofacial Pain and Dysfunction, Academic Centre for Dentistry of Amsterdam (ACTA), University of Amsterdam and Vrije Universiteit Amsterdam, Gustav Mahlerlaan 3004, 1081 LA Amsterdam, The Netherlands; 2 Department of Preventive Dentistry, Academic Centre for Dentistry of Amsterdam (ACTA), University of Amsterdam and Vrije Universiteit Amsterdam, Amsterdam, The Netherlands; 3 Department of Periodontology, Academic Centre for Dentistry of Amsterdam (ACTA), University of Amsterdam and Vrije Universiteit Amsterdam, Amsterdam, The Netherlands; 4 Amsterdam Rheumatology and Immunology Centre, locations Reade and Amsterdam University Medical Centre, Amsterdam, The Netherlands

**Keywords:** Quality of life, Oral health, Temporomandibular disorders, Pain, Periodontal health, Xerostomia

## Abstract

**Objectives:**

To evaluate oral health-related quality of life (OHRQoL) in early rheumatoid arthritis (ERA) patients and individuals at risk of rheumatoid arthritis (RA) compared to healthy controls, and to explore possible associated factors.

**Materials and methods:**

Fifty ERA patients, 50 at-risk individuals, and 50 age and gender matched healthy controls were recruited. OHRQoL (Oral Health Impact Profile-14 (OHIP-14)); number of decayed, missing, and filled teeth (DMFT); denture use; periodontal inflamed surface area (PISA); xerostomia (xerostomia inventory (XI)); and possible TMD (-pain) diagnoses were recorded. The groups were compared on these variables. Subsequently, backward multiple regression analyses were performed for the ERA and at-risk groups, with OHRQoL as the dependent variable and gender, age, DMFT, denture use, PISA, XI, non-painful TMD, and TMD pain as independent variables.

**Results:**

At-risk individuals had higher XI scores (U = 789.5, z = -3.181, p = 0.001, r = -0.32) and higher prevalence of TMD pain (p = 0.046, OR = 4.57; 95% CI 0.92–22.73) than healthy controls and higher OHIP-14 scores than the ERA group (U = 894.5, z = -2.418, p = 0.016, r = -0.24), while no difference in OHIP-14 was found between the control group and both other groups. For ERA patients, OHRQoL was associated with PISA and TMD pain (R^2^ = 0.498, p < 0.001). For at-risk individuals, OHRQoL was associated with XI score (R^2^ = 0.410, p < 0.001).

**Conclusions:**

Alertness of health professionals to TMD pain and periodontal inflammation in ERA patients and to xerostomia and TMD pain in at-risk individuals is recommended.

**Clinical relevance:**

The results of this study address orofacial aspects that require attention of health professionals in the timeframe around RA onset.

**Trial registration:** Dutch National Trial Register (NTR, NTR6362)

## Background

Rheumatoid arthritis (RA) is a chronic inflammatory disease of the joints that causes pain and can result in functional disability and in lower health-related quality of life (HRQoL) [1]. Since pharmacological treatment can alleviate symptoms and even prevent joint destruction, there is international consensus on the importance of early identification and treatment [2]. Several characteristics of RA, e.g., arthralgia and the presence of specific autoantibodies, can already be present before the development of clinical arthritis, and thus individuals at risk of RA can be identified [3].

While HRQoL covers a broad scope, the OHRQoL (oral health-related quality of life) reflects the subjective perception of local orofacial conditions on quality of life [4]. Like HRQoL, OHRQoL is a valuable patient-reported outcome measure (PROM) that provides insight into subjective disease burden. Current literature describes an association between RA and several orofacial aspects, e.g., periodontitis, xerostomia, and temporomandibular disorders (TMD) [5–8]—all of which can negatively influence the OHRQoL [9–14]. In addition, other orofacial aspects, e.g., the number of decayed, missing, and filled teeth (DMFT), and use of a denture, may affect OHRQoL [15]. Although previous studies do show a lower OHRQoL in patients with RA compared to healthy controls, only limited data is available on the timeframe around RA onset [16].

The importance of PROMs in RA is increasingly recognized in research and care [17–19], making QoL a relevant health outcome. Information on OHRQoL and possible associated orofacial conditions, in early RA (ERA) and in individuals at risk of RA, could highlight orofacial aspects that require targeted treatment. Hence, such information could provide a direction for customized care to limit orofacial inconveniences during an important timeframe. The aim of this study is therefore to evaluate the OHRQoL in patients with ERA and at-risk individuals compared to health controls, and to explore possible associated orofacial factors. We hypothesize a higher prevalence of periodontal disease, xerostomia, and TMD in ERA patients, and consequently lower OHRQoL, compared to healthy controls. Like other characteristics of RA, orofacial inconveniences might already occur before RA onset, and thus similar results are expected for the at-risk individuals.

## Methods

### Study design and ethical approval

This cross-sectional study is part of a larger longitudinal cohort study. A full description of the study protocol has been published previously [20]. The study protocol has been approved by the accredited Medical Ethical Committee of the Slotervaart Hospital and Reade (METc Slotervaartziekenhuis en Reade, U/17.056/P1719) and has been registered in the Dutch National Trial Register (NTR, NTR6362).

### Participants and recruitment

Three groups of participants were recruited: (1) patients with ERA, (2) individuals at risk of RA, and (3) a control group with no autoimmune conditions. Groups 1 and 2 were recruited at Reade, a rheumatology clinic in Amsterdam, the Netherlands. Group 1 consisted of patients diagnosed with RA according to the 2010 ACR/EULAR RA criteria [2] within the previous year. For group 2, participants were recruited from the Reade at-risk cohort [21–23]. Participants in this cohort have the combination of inflammatory-type arthralgia and increased serum levels of rheumatoid factor (RF) and/or anti-citrullinated protein antibodies (ACPA). For the current study, most participants in group 2 were included on the same day as inclusion in the Reade at-risk cohort, with a few exceptions of up to a maximum of 6 months hereafter. Participants for group 3 were recruited at the Academic Centre for Dentistry Amsterdam (ACTA), irrespective of oral status, and were matched to groups 1 and 2 regarding sex and age (± 5 years). All participants were ≥ 18 years, had a minimum of 12 natural teeth, and gave written informed consent. Venous blood was collected from all participants to determine serum levels of RF and ACPA, including participants in the control group to rule out a possible unknown increased risk of RA. Individuals with RF levels of > 5.0 kU/l and/or ACPA levels of > 10.0 kU/l were considered seropositive. All clinical examinations were performed by a single, experienced dentist (JMK).

### Outcome variables

#### Oral health-related quality of life

The shortened Oral Health Impact Profile (OHIP-14) questionnaire was used to measure OHRQoL [4]. The OHIP-14 is derived from the original 49-item OHIP, of which a Dutch translation has been developed and validated [24], and has been reported adequate for replacing the original 49-item OHIP [25]. With the OHIP-14, the frequency of a variety of possible social impacts of oral disorders (14 questions) during the past month is scored on a 5-point Likert scale, ranging from 0 (“never”) to 4 (“very often)”, resulting in a sum score ranging from 0 (no impact) to 56 (maximum impact of the oral health on quality of life).

#### Oral status

Participants were asked if they currently experienced intra-oral pain. Participants were also asked if they experienced any difficulties when performing oral hygiene during the past week, on a 4-point Likert scale ranging from 0 (“without any difficulty”) to 3 (“unable to do”). In case of a score other than 0, a further explanation was asked to identify possible joint problems related to RA. Data were analyzed as either having no difficulties (score 0) or having difficulties (score 1 to 3) performing oral hygiene.

An intra-oral inspection was performed to determine the total number of teeth present, and the number of decayed, missing, and filled teeth (DMFT) [26], using the International Caries Detection and Assessment System (ICDAS) score [27] of three or more as a cut-off value for caries presence, comparable to the WHO caries criteria [28]. The presence or absence of a removable (partial) denture was also recorded. Intra-oral soft tissues were inspected to detect possible mucosal abnormalities, e.g., wounds, abscesses, or fistulas.

#### Periodontal health

A periodontal examination included registration of bleeding on probing (BOP, present/absent), pocket probing depth (PPD) in millimeters, and positive gingival recession in millimeters on six sites for each tooth (mesiobuccal, midbuccal, distobuccal, mesiolingual, midlingual, and distolingual). Recording of BOP resulted in a full mouth BOP percentage. The total periodontal inflamed surface area (PISA) was calculated using the method described by Nesse et al. [29], to quantify the total burden of periodontal inflammation.

#### Xerostomia

To measure subjective symptoms of dry mouth, all participants were asked to fill in the xerostomia inventory (XI) questionnaire [30]. The XI is validated in several populations, including a Dutch population, with a forward-back-translation to validate the Dutch translation of the original questionnaire [31, 32]. The XI contains 11 questions about the frequency in which someone had to act on, or had trouble functioning because of, the adverse consequences of xerostomia during the past 4 weeks. All questions are scored on a 5-point Likert scale, ranging from 1 (“never”) to 5 (“very often”), resulting in a sum score ranging from 11 (no dry mouth) to 55 (extremely dry mouth).

#### Temporomandibular disorders

A thorough description of the methods and results concerning classification of possible TMD has been previously described [33]. In brief, the presence of TMD was classified according to the Diagnostic Criteria for TMD (DC/TMD) [34], and five diagnostic categories were recognized: (1) myalgia, (2) arthralgia, (3) disc displacement, (4) degenerative joint disease, and (5) headache attributed to TMD. All participants filled out the DC/TMD symptom questionnaire with 14 questions on pain in the joint area, headache, joint sounds, and joint locking [35]. The clinical examination was performed according to the DC/TMD Clinical Examination Protocol [36]. Results were analyzed for having non-painful TMD diagnoses (disc displacement, degenerative joint disease, or both) and for having TMD-pain diagnoses (myalgia, arthralgia, or both). To be diagnosed with a headache attributed to TMD, a myalgia and/or arthralgia diagnosis is required, and thus this category was not separately analyzed.

### Statistical analysis

Characteristics of the study population were described using descriptive statistics. First, normal distribution of variables was tested with the Kolmogorov–Smirnov test. For normally distributed variables, the mean (and standard deviation (SD)) is reported; otherwise, the median (and interquartile range (IQR)) is reported. For normally distributed continuous variables, one-way ANOVA with post hoc independent samples t-tests was used when comparing the means of the three groups; otherwise, the Kruskal–Wallis test was used, with post hoc Mann–Whitney U tests. Differences between groups on binary variables were tested with a Chi-square test or Fisher’s exact test when appropriate. Probability levels of less than 0.05 were considered statistically significant.

Subsequently, to evaluate possible associations between OHRQoL and orofacial conditions, a backward multiple linear regression analysis was performed for the ERA group and the at-risk group. The decision to use this type of analysis was based on the advantage of considering the possible effects, and importance of these effects, of all variables simultaneously. The analysis used OHRQoL as the dependent variable and the following independent variables: gender, age, DMFT, use of a removable (partial) denture, PISA, XI, non-painful TMD, and TMD pain. First, as a preselection procedure, the unadjusted associations with the independent variables were tested. Variables that showed at least a weak association (p < 0.10) were then included in the multiple regression model. A backward approach was used, where the variables with the weakest predictive value were removed step-by-step, until all independent variables showed at least a p value < 0.05 in the final model. From the regression analyses, significant associated factors and their correlation coefficients were extracted.

Variables were tested for multicollinearity based on the variance inflation factor (VIF), and all VIF values were between 1 and 5 and thus considered inconsequential correlations [37]. All analyses were performed using the IBM SPSS Statistics 26 software package (IBM Corp, Armonk, NY, USA).

## Results

### Sample demographics and orofacial variables

From November 2017 until July 2019, a total number of 150 participants were included, 50 per group. Unless specifically described otherwise below, data on the measured variables were available for all 150 participants. Table 1 displays the characteristics of the study population. There was no difference in average age or gender distribution between the groups. In the ERA group, patients were included an average of 3.1 ± 1.7 months after being diagnosed with RA.

**Table 1:** Demographics of the study sample and results on dental status, periodontal health, xerostomia, and oral health-related quality of life

	ERA group (n = 50)	At-risk group (n = 50)	Control group (n = 50)	*p*	Post hoc*, p*
Age, years [mean (SD)]	52.1 (13.2)	51.4 (10.3)	51.2 (11.0)	NS^1a^	
Gender, female [*n* (%)]	39 (78%)	38 (76%)	38 (76%)	NS^1b^	
RF or ACPA positive [*n* (%)]	38 (76%)	50 (100%)	0 (0%)	N/A	
Dental status					
Total number of teeth [median (IQR)]	27.0 (24.8–28.0)	27.0 (25.0–28.0)	27.5 (25.0–28.0)	NS^1c^	
DMFT [mean (SD)]	12.8 (7.0)	12.2 (5.9)	11.0 (6.5)	NS^1a^	
Removable (partial) denture [*n* (%)]	4 (8%)	4 (8%)	3 (6%)	NS^1b^	
Intra-oral pain [n (%)]	10 (20%)	11 (22%)	4 (8%)	NS^1b^	
Difficulties with oral hygiene [*n* (%)]	4 (8%)	8 (16%)	2 (4%)	NS^1b^	
Periodontal health					
BOP (%) [median (IQR)]	19.3 (9.9–35.4)	15.4 (7.4–32.5)	17.5 (8.5–27.5)	NS^1c^	
Average PPD, mm [median (IQR)]	2.2 (2.0–2.6)	2.2 (2.0–2.5)	2.1 (1.9–2.5)	NS^1c^	
PISA, mm^2^ [median (IQR)]	258.3 (108.4–398.3)	191.3 (72.5–431.8)	181.4 (91.2–369.4)	NS^1c^	
Xerostomia					
XI score [median (IQR)]	19 (16–25)	22 (17–27)	17 (13–21)	*0.005*^1c^	0.11^2**e**^, 0.81^3**e**^, *0.001*^4**e**^

*ERA* early rheumatoid arthritis, *n* number of observations, *SD* standard deviation, *RF* rheumatoid factor, *ACPA* anti-citrullinated protein antibodies, *IQR* Interquartile range, *DMFT* decayed missing and filled teeth, *BOP* bleeding on probing, *PPD* pocket probing depth, *PISA* periodontal inflamed surface area, *XI* xerostomia inventory. Significant results are shown in italicized font (p < 0.05)

^a^One-way ANOVA, ^b^Chi-square test, ^c^Kruskal-Wallis, ^d^Fisher’s exact, ^e^Mann-Whitney U

^1^Three groups, ^2^ERA group versus at-risk group, ^3^ERA group versus control group, ^4^At-risk group versus control group

### Oral status and periodontal health

No significant differences were found between the three groups on number of teeth present, DMFT, prevalence of using a removable (partial) denture, or prevalence of currently present intra-oral pain (Table 1). The reported intra-oral pain was of dental origin in 15 cases, of periodontal origin in seven cases, and originating from the intra-oral soft tissues in three cases. Due to the overlap with DMFT and periodontal health, the currently present intra-oral pain variable was not added to the regression analyses.

There also was no difference between the groups in number of participants that reported difficulties with performing oral hygiene during the past week due to physical complaints in joints of the hands and/or arms (Table 1). Interestingly, an additional five participants in the ERA group reported that they used to have difficulties with performing oral hygiene, but this was resolved after starting with the pharmacological treatment for RA.

Further, no differences were found for the investigated periodontal variables, i.e., BOP, average PPD, and PISA (Table 1).

### Xerostomia

Forty-nine participants in the at-risk group, and all participants in the ERA group and control group completed the XI questionnaire. The median total XI score of the at-risk group was significantly higher, indicating more subjective xerostomia, compared to the control group (U = 789.5, z = -3.181, p = 0.001, r = -0.32) (Table 1). No difference was found between the ERA group and the other two groups (Table 1).

### Temporomandibular disorders

**Fig. 1:**
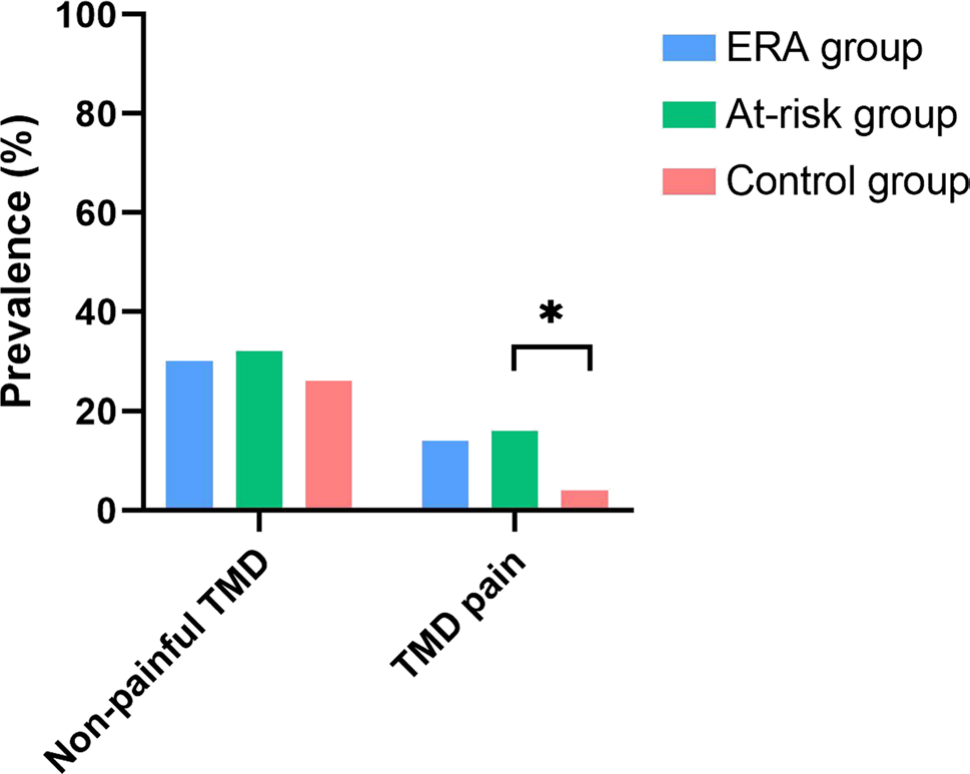
Prevalence of non-painful TMD and TMD pain in patients with early rheumatoid arthritis (ERA), individuals at risk of RA, and healthy controls. An asterisk indicates a significant difference

A thorough description on results for TMD (pain) in the study population has been previously reported [33]. A summary of the results is shown in Fig. 1. In brief, the three groups did not differ when comparing them on the total number of TMD diagnoses or when comparing them on non-painful TMD diagnoses. However, when considering TMD-pain diagnoses only—either myalgia, arthralgia, or both—participants in the at-risk group more often received a TMD-pain diagnosis than those in the control group (p = 0.046, OR = 4.57; 95% CI 0.92–22.73). No difference was found between the ERA group and the control group.

#### Oral Health Related Quality of Life

**Fig. 2:**
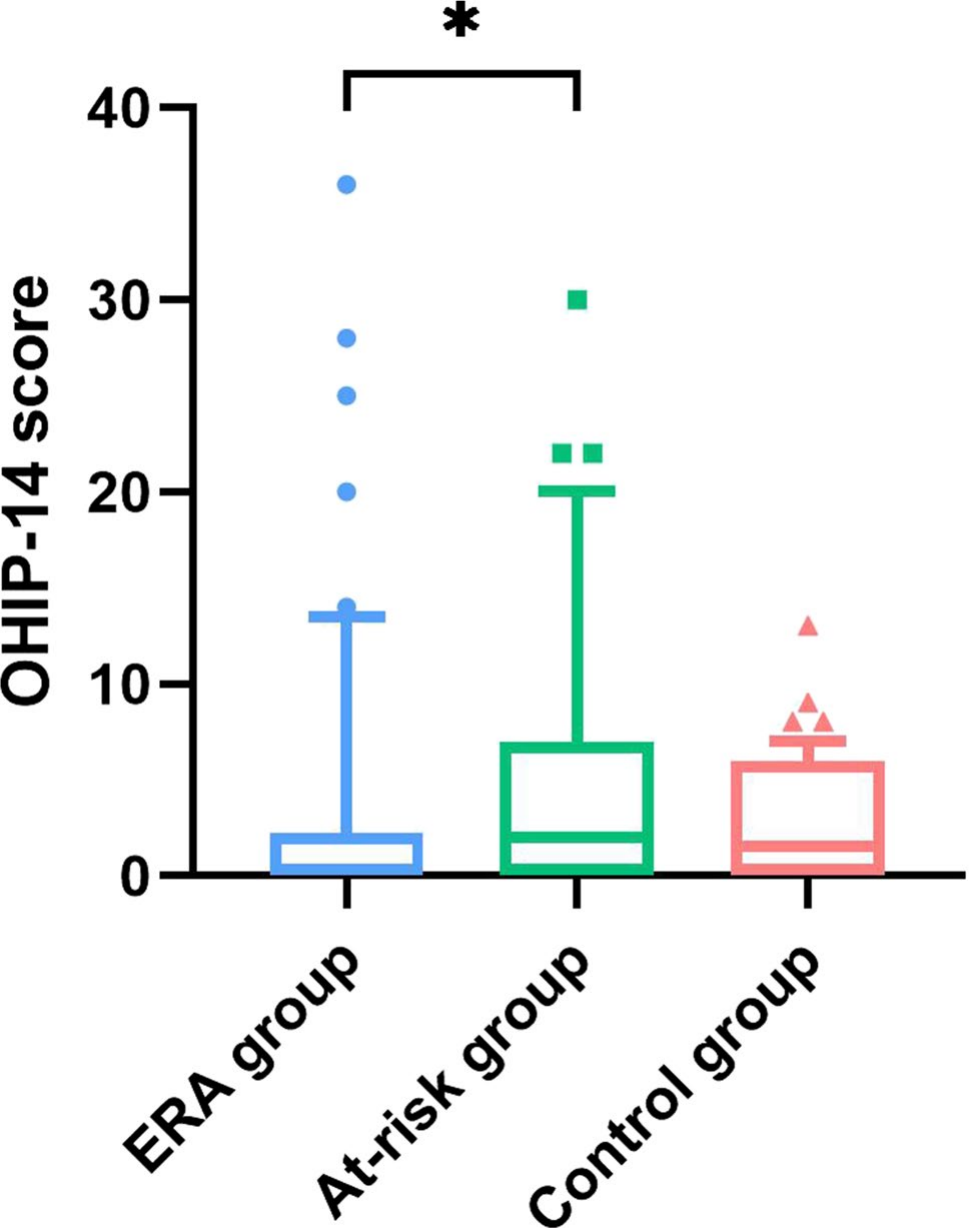
Oral health-related quality of life (OHRQoL) according to the OHIP-14 score in patients with early rheumatoid arthritis (ERA), individuals at-risk of RA, and healthy controls. A Kruskal–Wallis test showed a significant result (p = 0.042). Post hoc Mann–Whitney U tests showed a significant result between the ERA group and at-risk group (p = 0.016, indicated by an asterisk), but not between the ERA group and at-risk group compared to the control group (p = 0.116 and p = 0.248, respectively)

Forty-nine participants in the ERA group and all participants in the at-risk group and the control group completed the OHIP-14 questionnaire. Distributions of the total OHIP-14 scores per group are shown in Fig. 2. The median OHIP-14 score of the at-risk group was significantly higher, indicating lower OHRQoL, compared to the ERA group (U = 894.5, z = -2.418, p = 0.016, r = -0.24). No difference was found between the ERA group or at-risk group compared to the control group (p = 0.116 and p = 0.248, respectively).

To explore potential factors that were associated with OHRQoL, we performed further analyses. The results of the single and multiple linear regression analyses on OHRQoL for the ERA group and at-risk group are shown in Tables 2 and 3, respectively. In the ERA group, using a removable (partial) denture, XI score, PISA, and TMD pain showed at least a weak association (p < 0.10) with the OHIP-14 score during the preselection. In the backward multiple regression analysis, only PISA (p < 0.001) and TMD pain (p < 0.001) remained significant. The model predicted 49.8% of the outcome, and both PISA and TMD pain showed a positive correlation to the OHIP-14 score. This means that both an increase in PISA and presence of TMD pain result in a higher OHIP-14 score, and thus lower OHRQoL. The standardized beta suggests a slightly larger effect of TMD pain than of PISA on QoL.

**Table 2:** Association of orofacial conditions with oral health-related quality of life (OHRQoL) in the group of patients with early rheumatoid arthritis (ERA) (n = 50)

	Single regression (preselection)	Backward multiple regression				
Independent variable	*p* Value	p-to-exit Value	*p* Value	Regression coefficient	95% CI	Standardized Beta
Gender (f/m)	0.975					
Age (years)	0.176					
DMFT	0.367					
Use of removable (partial) denture (y/n)	*0.060*	0.123				
PISA	< *0.001*		< *0.001*	0.012	0.006–0.017	0.445
XI	*0.016*	0.140				
Non-painful TMD	0.485					
TMD pain	< *0.001*		< *0.001*	11.456	6.791–16.121	0.518

*OHRQoL* oral health related quality of life, *ERA* early rheumatoid arthritis, *DMFT* decayed missing and filled teeth, *PISA* periodontal inflamed surface area, *XI* xerostomia inventory, *TMD* temporomandibular disorders, *CI* confidence interval. Significant results are shown in italicized font (p < 0.10 in preselection, p < 0.05 in multiple regression)

Multiple regression: R^2^ = 0.498, p < 0.001

**Table 3:** Association of orofacial conditions with oral health-related quality of life (OHRQoL) in the group of individuals at risk of developing rheumatoid arthritis (RA) (n = 49)

	Single regression (preselection)	Backward multiple regression				
Independent variable	*p* Value	p-to-exit Value	*p* Value	Regression coefficient	95% CI	Standardized Beta
Gender (f/m)	*0.045*	0.665				
Age (years)	*0.051*	0.203				
DMFT	0.71					
Use of removable (partial) denture (y/n)	0.772					
PISA	0.207					
XI	< *0.001*		*0.001*	0.525	0.340–0.710	0.641
Non-painful TMD	0.641					
TMD pain	< *0.001*	0.087				

*OHRQoL* oral health related quality of life, *RA* rheumatoid arthritis, *DMFT* decayed missing and filled teeth, *PISA* periodontal inflamed surface area, *XI* xerostomia inventory, *TMD* temporomandibular disorders, *CI* confidence interval. Significant results are shown in italicized font (p < 0.10 in preselection, p < 0.05 in multiple regression)

Multiple regression: R^2^ = 0.410, p < 0.001

In the at-risk group, sex, age, XI score, and TMD pain showed at least a weak association (p < 0.10) with the OHIP-14 score during the preselection. However, according to the backward multiple regression model, only the XI score significantly predicted the OHIP-14 score (p = 0.001) in this population. The model predicted 41% of the outcome.

## Discussion

Participants in the at-risk group experienced lower oral health-related quality of life (OHRQoL) compared to the patients in the early rheumatoid arthritis (ERA) group. Within the at-risk group, xerostomia was the only variable that was associated with OHRQoL, with an increase in xerostomia resulting in lower OHRQoL. Furthermore, the median xerostomia index (XI) score was higher, indicating more xerostomia, in the at-risk group compared to the control group. Although challenging to prevent or treat, these results indicate that health professionals should be alert to xerostomia complaints in individuals at risk of RA.

Since the prevalence of TMD pain in the at-risk group was significantly higher than in the control group, alertness to TMD pain in at-risk individuals is also recommended, as previously described [33]. Although the single regression analysis showed a strong association between TMD pain and OHRQoL in the at-risk group, TMD pain was excluded from the multiple regression model. This might be caused by the effect of xerostomia eliminating the effect of TMD pain when considering all variables simultaneously. However, due to the low number of participants further discussed in the limitations below, the possibility that insufficient data was available to identify a significant effect of TMD pain in the multiple regression model should also be considered.

For the ERA group, lower OHRQoL was associated with TMD pain and higher periodontal inflamed surface area (PISA). Both prevalence of TMD pain and that of PISA were not higher compared to the control group. However, the negative effect on OHRQoL suggests that both TMD pain and periodontal inflammation require attention in some patients. Screening for both conditions in patients with ERA could lead to timely treatment and, consequently, alleviation of discomfort.

Interestingly, the results of this study indicate different associated factors for OHRQoL in ERA patients and at-risk individuals. TMD pain was found to be significantly associated with OHRQoL in ERA patients, while in the at-risk group, the multiple regression analysis did not identify TMD pain as a significant influence on OHRQoL. In both groups, non-painful TMD was not associated with OHRQoL, indicating that only painful TMD negatively influences QoL.

Further, while PISA was found to be an associated factor in ERA, it did not pass the preselection for the regression analysis in at-risk individuals. Although not significant, numbers on periodontal variables were higher for ERA patients compared to both the at-risk group and the control group, possibly resulting in a more profound effect on OHRQoL.

Contrarily, XI score was found to be significantly associated with OHRQoL in at-risk individuals, but not in ERA patients. The XI is intended as a continuous scale to reduce the risk of misclassification error by using an arbitrary cut-off [30]. However, in a study where both subjective and objective dry mouth were measured, the median XI score in people with normal salivation was reported to be 22.5, while for low salivation and hyposalivation, median XI scores of 25 and 39, respectively, were reported [38]. Considering these median scores, XI scores in the current study population are overall relatively low, and prevalence of clinically relevant xerostomia in the ERA group might thus be too low to detect an association with OHRQoL.

Previous studies found poorer dental status and periodontal health in patients with RA compared to healthy controls [6, 39, 40]. The current study cannot confirm these results, possibly because only RA patients that were very early in the disease were included. Poorer oral health could be caused by impaired oral hygiene due to RA disease activity in the joints of the hands, but the results of this study show little self-reported difficulty in performing oral hygiene. A relation between systemic RA inflammation and inflammation of the periodontal tissues has also been suggested [41, 42]. It is imaginable that the effects are not yet present during the first few months since diagnosis, because tooth decay and chronic periodontal inflammation usually require more time to develop. Furthermore, most ERA patients received pharmacological treatment for RA, which can have a positive effect on periodontal inflammation [42]. Our results also indicate a positive effect of pharmacological treatment on oral hygiene, since five ERA patients reported resolving of their difficulties with performing oral hygiene since the start of the pharmacological treatment for RA.

### Strengths and limitations

This study reports on OHRQoL and associated factors in a very specific population within the timeframe around RA onset, i.e., patients with ERA and individuals at risk of RA. Consequentially, it required a considerable amount of time to include 50 participants per group. For regression analyses, approximately ten participants are required for each independent variable that is tested. Therefore, a preselection was performed before building the multiple linear regression model. Although a total number of 50 participants per group is thus marginal, it was enough considering the number of variables that were eventually entered into the model. The relatively low number of participants and overall low median OHIP-14 scores, which correspond to OHRQoL in the general Dutch population [43] and a group of type 2 diabetes patients in the Amsterdam area [44], do imply that results are based on a limited number of participants with a high OHIP-14 score and thus low OHRQoL.

In RA patients with longer disease duration, OHRQoL was found to be lower than in healthy controls [16], but the current study cannot confirm this for patients early in the disease. This possibly is because also no differences were found in dental status, periodontal inflammation, TMD pain, and xerostomia—all of which could lower OHRQoL—compared to the healthy control group. It is also imaginable that the burden of a newly diagnosed general disease, and consequently doctor visits and medication, could overshadow orofacial inconveniences.

Further, the XI score was used to measure xerostomia, which only illustrates the subjective perception of dry mouth. For future research, clinical measurement could add valuable information on objective dry mouth, for example, by measuring salivary flow rate and intra-oral examination using the Clinical Oral Dryness Scale [38].

## Conclusion

No difference was found when comparing the OHRQoL of ERA patients and at-risk individuals to healthy controls. Although periodontal inflammation and TMD pain were not more prevalent in ERA patients compared to healthy controls, they do negatively influence the OHRQoL, and screening by health professionals for both conditions is thus recommended. In individuals at risk of RA, alertness to xerostomia and TMD pain is recommended, since prevalence of both conditions is higher compared to healthy controls, and xerostomia negatively influences the OHRQoL.
